# Functional Assessment for Clinical Use of Serum-Free Adapted NK-92 Cells

**DOI:** 10.3390/cancers11010069

**Published:** 2019-01-10

**Authors:** Michael Chrobok, Carin I. M. Dahlberg, Ece Canan Sayitoglu, Vladimir Beljanski, Hareth Nahi, Mari Gilljam, Birgitta Stellan, Tolga Sutlu, Adil Doganay Duru, Evren Alici

**Affiliations:** 1 Center for Hematology and Regenerative Medicine, Department of Medicine Huddinge, Karolinska Institutet, 141 83 Stockholm, Sweden; Michael.Chrobok@ki.se (M.C.); Carin.Dahlberg@ki.se (C.I.M.D.); Hareth.Nahi@ki.se (H.N.); Mari.Gilljam@ki.se (M.G.); birgittastellan@gmail.com (B.S.); 2 NSU Cell Therapy Institute, Nova Southeastern University, Fort Lauderdale, FL 33314, USA; esayitoglu@nova.edu (E.C.S.); vbeljanski@nova.edu (V.B.); adil.duru@nova.edu (A.D.D.); 3 Haematology Centre, Karolinska University Hospital, 141 57 Huddinge, Sweden; 4 Nanotechnology Research and Application Center, Sabanci University, 34956 Istanbul, Turkey; tolgasutlu@sabanciuniv.edu

**Keywords:** NK-92, NK cell, serum-free, immunotherapy

## Abstract

Natural killer (NK) cells stand out as promising candidates for cellular immunotherapy due to their capacity to kill malignant cells. However, the therapeutic use of NK cells is often dependent on cell expansion and activation with considerable amounts of serum and exogenous cytokines. We aimed to develop an expansion protocol for NK-92 cells in an effort to generate a cost-efficient, xeno-free, clinical grade manufactured master cell line for therapeutic applications. By making functional assays with NK-92 cells cultured under serum-free conditions (NK-92_SF_) and comparing to serum-supplemented NK-92 cells (NK-92_S_) we did not observe significant alterations in the viability, proliferation, receptor expression levels, or in perforin and granzyme levels. Interestingly, even though NK-92_SF_ cells displayed decreased degranulation and cytotoxicity against tumor cells in vitro, the degranulation capacity was recovered after overnight incubation with 20% serum in the medium. Moreover, lentiviral vector-based genetic modification efficiency of NK-92_SF_ cells was comparable with NK-92_S_ cells. The application of similar strategies can be useful in reducing the costs of manufacturing cells for clinical use and can help us understand and implement strategies towards chemically defined expansion and genetic modification protocols.

## 1. Introduction

Natural killer (NK) cells are potential candidates for adoptive immunotherapy against cancer [1]. NK cells from different sources have been utilized in various clinical trials with a robust safety profile and varying degrees of success [1,2]. As an alternative to primary NK cells, the possibility of using cytotoxic cell lines as adoptive immunotherapy has also been investigated [3,4,5,6,7]. One of the advantages of using cytotoxic cell lines is the possibility of producing large amounts of clonal and well-defined effector cell populations. To our knowledge, the only available NK cell line that has been tested in clinical trials is the NK-92 cell line which was generated by Hans Klingemann in 1992 [5,8,9]. NK-92 originates from non-Hodgkin’s Lymphoma cells derived from a 50-year old male patient [5]. NK-92 cells express high levels of activating receptors, such as NKG2D, NKp30, NKp46, and 2B4 [10], and are highly cytotoxic against a broad range of tumor cells [11,12,13,14]. This may be due to their lack of almost all inhibitory killer-immunoglobulin-like receptors (KIRs) [10], which results in impaired inhibitory signaling triggered by a lack of KIR/HLA interactions (missing-self) [15]. NK-92 cells have been used in several published clinical trials targeting both solid and hematological malignancies. The two latest published clinical trials with the NK-92 cell line treated several hematological malignancies, such as acute myeloid leukemia [16], multiple myeloma, and Hodgkin’s lymphoma [17]. In the published clinical trials, no severe infusion-related or long-term side effects were observed and promising anti-tumor effects were observed in some patients [8,9,16,17]. Several clinical trials are ongoing with genetically modified NK-92 cells. In 2018, the first CD33-chimeric antigen receptor (CAR) NK-92 cell line infusion was published [18]. Three patients with relapsed or refractory acute myeloid leukemia AML were safely infused with genetically modified NK-92 cells but without obvious clinical efficacy [18]. Even though the results in clinical trials are promising, a unified protocol of expanding NK-92 cells for clinical application has not yet been established. To our knowledge, in all clinical trials that have been conducted on patients with advanced cancer progression, the NK-92 cells have been cultured in a high percentage of plasma.

To optimally manufacture NK-92 cells for clinically relevant doses, one of the remaining challenges is the need for serum, either from a donor pool or from a xenogeneic source. The original standard culturing conditions for the NK-92 cell line contains 12.5% fetal bovine serum (FBS) and 12.5% horse serum [19], a combination that is assumed to be necessary for the growth of this cell line [19]. Sera contain a large number of constituents, low and high molecular weight biomolecules, like proteins, hormones, growth factors and minerals with different physiological growth-promoting, and growth-inhibitory factors [20]. As endotoxins are present in all sera, but with levels highly fluctuating between different batches, it is important to validate different batches. For optimal cell growth, it is crucial to keep the endotoxin levels low. This, together with the fluctuation of serum-containing components between different sera batches leads to a high variability of cell proliferation and, thus, an unpredicted outcome of cell expansion protocols.

In this study, we investigated the possibility to overcome serum dependency of the NK-92 cell line, by gradually adapting these cells to serum-free culturing conditions. Furthermore, we aimed to compare effector functions and the transcriptional profile as well as the proliferative capacity and the susceptibility to genetic modifications using lentiviral vectors.

## 2. Results

### 2.1. NK-92_SF_ Cells Retained Colony Formation Capacity and High Viability

**Figure 1:**
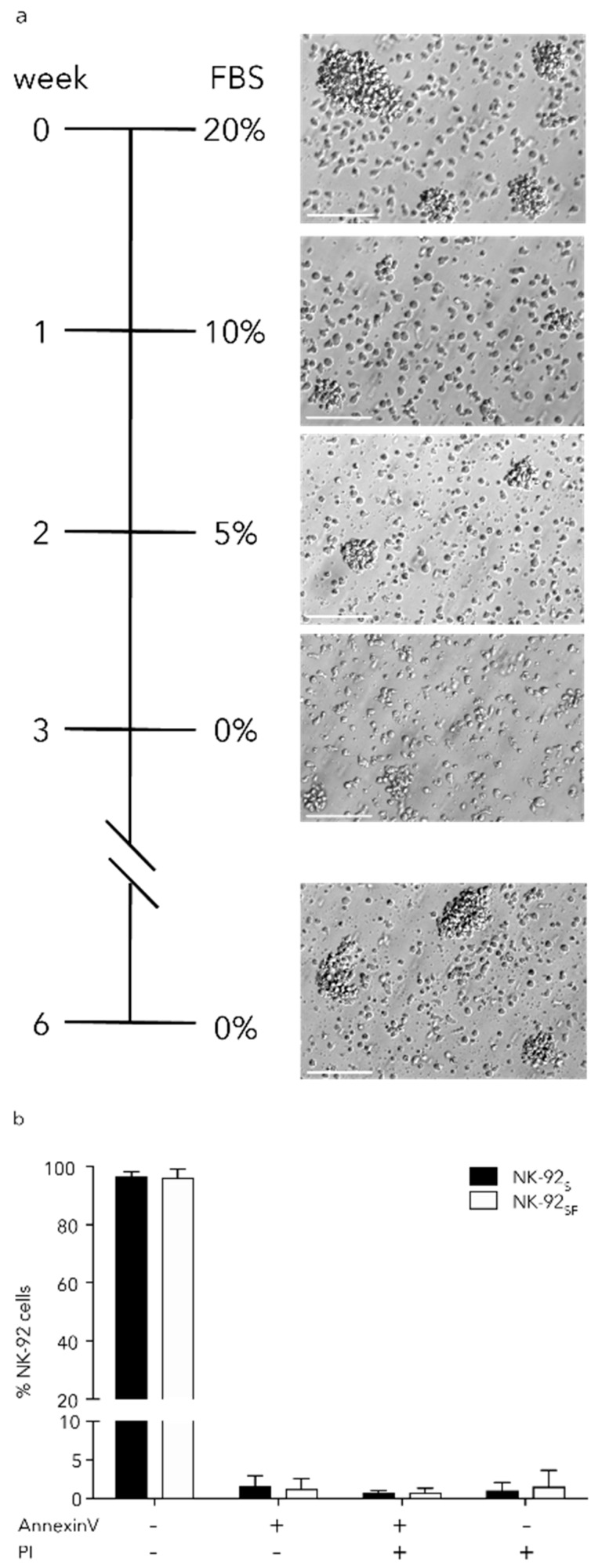
Serum-free culture of NK-92 cells does not affect colony formation and viability. (**a**) Schematic overview of the serum starvation process over four weeks following a three-week recovery phase. Microscope images of NK-92 cells during the starvation process. Scale bar equals 100 μm. (**b**) Staining with Annexin V and propidium iodide (PI) of CD56^+^ NK-92 cells, after the 3-week recovery period, identified live cells (Annexin V^−^/PI^−^), pre- (Annexin V^+^/PI^−^) and pro-apoptotic (Annexin V^+^/PI^+^) cells as well as necrotic cells (Annexin V^−^/PI^+^). Each bar represents the mean (+SD) of eight independent assays performed in duplicates.

NK-92 cells are cultured in Good Manufacturing Practice (GMP)-grade, serum-free, xeno-free stem cell growth medium (CellGro SCGM) supplemented with 20% FBS and 1000 U/mL Proleukin or IL-2 in our laboratory. We used a serum reduction strategy in which serum levels were gradually reduced from 20% to 0% over a 4-week period which equals approximately 12 passages to obtain serum-free cultured NK-92 cells (NK-92_SF)_ (Figure 1a).

Initially, we observed a transient decrease of colonies formed by NK-92_SF_ cells when compared to NK-92 cells cultured with 20% FBS (NK-92_S_). However, the colony formation was restored six weeks after the serum reduction was initiated (Figure 1a). Comparative analysis of cell viability and necrotic cells (Annexin V^−^, propidium iodide^+^) revealed that there was no alteration in viability between NK-92_SF_ cells and NK-92_S_ cells (Figure 1b). All the following experiments have been performed between six weeks after initiation of serum reduction and four months of culture.

### 2.2. NK-92 Cells Proliferated in the Absence of Serum

**Figure 2:**
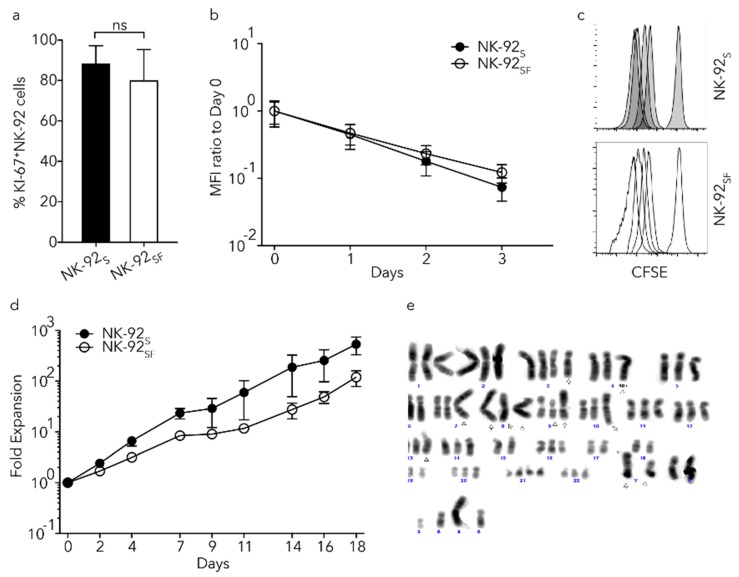
Proliferation of serum-free NK-92 cell culture is slightly reduced. (**a**) Staining of NK-92_S/SF_ cells for KI-67 as a measurement of proliferation. Each bar represents the mean (+SD) of eight independent assays performed in duplicates. (**b**) Cell division between NK-92_S_ and NK-92_SF_ cells was monitored for three days. Analyzed by comparing intracellular carboxyfluorescein succinimidyl ester (CFSE) dilution to day 0. Each data point represents the mean (±SD) of two independent assays performed in triplicates. (**c**) Representative CFSE histograms for four consecutive days. (**d**) Cell division of NK-92 cells was monitored for 18 days after the recovery phase and fold expansion was calculated compared to day 0. The graph shows one representative experiment where each data point represents the mean (±SD) of triplicates. (**e**) Representative karyotyping picture from NK-92_SF_ cells after the recovery phase. Arrowheads show previously published alterations of the chromosomes in NK-92 cells. Colors were inverted from the original image. (**a**): Welch’s *t*-test was used.

Since no differences in viability were observed in the NK-92_SF_ cells compared to NK-92_S_ cells, we next wanted to investigate the impact of serum reduction on the proliferative capacity of NK-92 cells. We could not detect differences in the percentage of proliferating cells between NK-92_SF_ cells and NK-92_S_ cells with Ki-67 staining (Figure 2a).

Cell proliferation analysis using carboxyfluorescein succinimidyl ester (CFSE) labeling proved that NK-92_SF_ cells have comparable mitotic rates to NK-92_S_ cells (Figure 2b,c). Since we could not detect any proliferation differences between NK-92_SF_ cells and NK-92_S_ cells during three days of CFSE measurements, we were interested in determining if the absence of serum affects long-term proliferation. NK-92_SF_ cells were expanded under standardized conditions for 18 days and did not show any large difference in proliferation rate (Figure 2d). In addition, we kept the cells in culture for over four months and could not observe a difference in proliferation capacity during this time period. Overall, NK-92 cells can be cultured in a serum-free SCGM medium without altering the viability; however, the proliferation capacity of NK-92_SF_ cells is slightly lower compared to NK-92_S_.

### 2.3. NK-92_SF_ Cells Showed Varying Karyotypes

The karyotype of NK-92 cells has been extensively investigated and described by H.G. Drexler [21]. The analysis of NK-92_S_ cells showed the same aberrations as described before [21]. We have observed that NK-92_SF_ cells acquired additional aberrations involving 4p in 10/20 metaphases (Figure 2e). In the second batch, among the 23 metaphases that were analyzed from NK-92_SF_ cells, no addition on chromosome 4 could be found. Within the second batch of NK-92_SF_ cells, 6/23 metaphases showed a del(1) and seven other metaphases an additional del12(p+). To conclude the karyotyping of NK-92_SF_ cells, we have not observed any reproducible genetic drift.

### 2.4. NK-92_SF_ Cells Showed Similar Receptor Expression Profile as NK-92_S_ Cells

**Figure 3:**
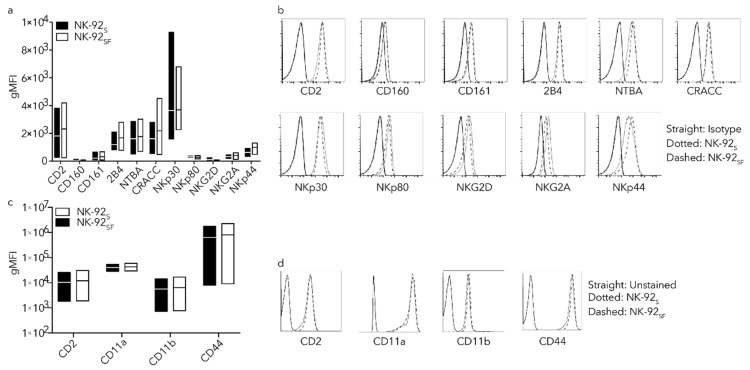
Receptor expression is not altered on NK-92_SF_ cells. NK-92 cells were analyzed with a full panel of NK-cell markers. Only markers with detected expression, i.e., higher than unstained samples, are shown in the figure. Geometric mean fluorescent intensity (gMFI) of NK cell markers activation markers (**a**) or adhesion markers (**c**) on NK-92_S_ and NK-92_SF_ cells. (**b**) Representative histograms showing indicated receptor expression. Straight line shows isotype control, dotted line shows receptor expression on NK-92_S_, and dashed line shows receptor expression on NK-92_SF_. (**d**) Representative histograms for adhesion receptors on NK-92 cells. Straight line shows unstained control, dotted line shows receptor expression on NK-92_S_ cells, and dashed line shows receptor expression on NK-92_SF_ cells. Floating bars show mean and min/max value of three independent assays performed as a single or duplicate experiment.

In order to characterize phenotypic changes in NK-92 cells after serum reduction, an NK cell receptor screening was performed using multi-parameter flow cytometry. For the screening we used antibody panels adapted from previous work published by our group [22]. NK cell markers that are known to be abundantly expressed, such as the natural cytotoxicity receptors (NCRs) NKp30, NKp44 and NKp46, as well as other activating receptors like 2B4, DNAM-1, NKG2D, and NKG2C have been analyzed by flow cytometry (Figure 3a,b). Many receptors were not expressed, and therefore not presented in Figure 3. In order to assess if the adhesion capacity of NK-92_SF_ cells were altered, we checked several adhesion markers. We tested expression of the following receptors: CD2, CD11a (LFA-1), CD11b, CD44, CD58, and CD62L (Figure 3c,d). The markers CD58 and CD62L could not be detected on NK-92 cells.

### 2.5. Altered Gene Expression of NK-92_SF_ Cells

**Figure 4:**
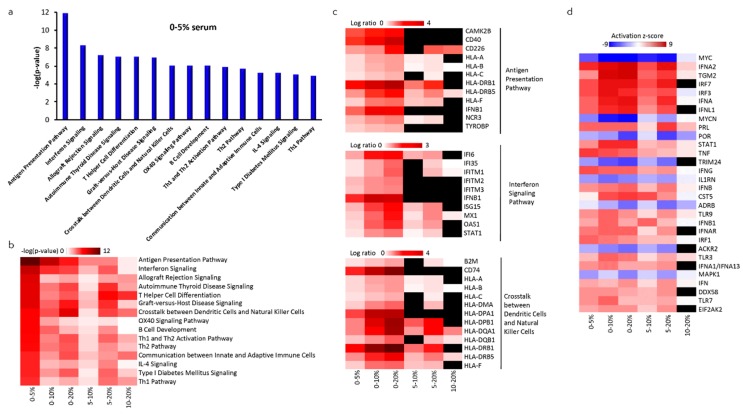
Analysis of gene expression profiles in NK-92 cells exposed to different concentrations of serum. (**a**) Canonical pathway analysis for NK-92 cells exposed to 5% serum compared to cells grown without serum; (**b**) the heatmap shows statistically significant differentially regulated pathways created by comparison of differential gene expression contrasts for contrasts indicated below; (**c**) differential gene expression for pathways related to immune response. Missing values that did not reach statistical significance criteria are shown in black. (**d**) Ingenuity Pathway Analysis (IPA) upstream transcriptional regulator analysis for genes that are found to be differentially regulated in datasets.

We performed RNA sequencing to generate gene expression profiles of NK-92 cells cultured in 5%, 10%, or 20% serum and compared this to NK-92_SF_ cells to investigate the biological mechanism(s) involved in the adaptation of NK-92 cells to serum reduction. Comparisons of gene expression profiles were subsequently generated and uploaded into Ingenuity Pathway Analysis (IPA). To further evaluate differentially regulated genes and pathways, we analyzed differences in expression profiles of genes belonging to the top 15 statistically significant pathways based on IPA that are unique to each serum condition (Figure 4a). Expression profile comparisons between cells grown in different serum conditions revealed changes in pathways associated with immune responses, and the most statistically significant changes were observed between serum-free cells and those grown in 5, 10, or 20% serum (Figure 4b). In the 0–5% serum comparison, 13 out of 38 genes (34.2%) from the antigen presentation pathway and 10 out of 36 genes (27.8%) from the interferon signaling pathway were upregulated (Figure 4a).

The most statistically significant changes in those pathways were associated with increased expression of major histocompatibility class I (A, B, C, F) and II genes (DM α, DP α and β 1, DQ α and β 1, DR β 1, and DR β 5) as well as interferon-stimulated genes (interferon-induced proteins (IFIT) 6 and 35, interferon-induced transmembrane proteins (IFITM) 1–3, interferon-stimulated gene 15 (ISG15), signal transducer and activator of transcription 1 (STAT1)) and were common for all six comparisons. A similar set of differentially regulated genes also contributed to the significant association of other pathways listed in Figure 4c. However, the number of differentially regulated genes decreased subsequent serum concentration increase (Figure 4c). This indicates that the pathways associated with immune response are not additionally stimulated by serum increase once serum is introduced already at low levels.

We also performed upstream analysis for all comparisons to identify the cascade of upstream transcriptional regulators that can explain the observed transcriptomic changes occurring during serum reduction. This upstream regulator analysis is based on prior knowledge of expected effects between transcriptional regulators and their target genes based on the IPA knowledge base. This analysis revealed down-regulation of V-Myc Avian Myelocytomatosis Viral Oncogene Homolog (MYC) and V-Myc Avian Myelocytomatosis Viral Oncogene Neuroblastoma Derived Homolog (MYCN)-driven signaling upon an increase in serum, indicating that NK-92_SF_ cells rely on MYC oncogene for survival (Figure 4d).

Overall, our data indicate that serum reduction induces changes in expression of genes that are involved in regulation of immune response, protein, and cholesterol biosynthesis pathways and are likely the most prominent biological responses induced by the addition of serum. Participation of such pathways changes based on the concentration of serum: Lower concentrations favor immune response pathways and higher concentrations favor cholesterol synthesis pathways.

### 2.6. NK-92_SF_ Cells Displayed Normal Expression of Cytotoxic Molecules but with Reduced Cytotoxic Capacity

**Figure 5:**
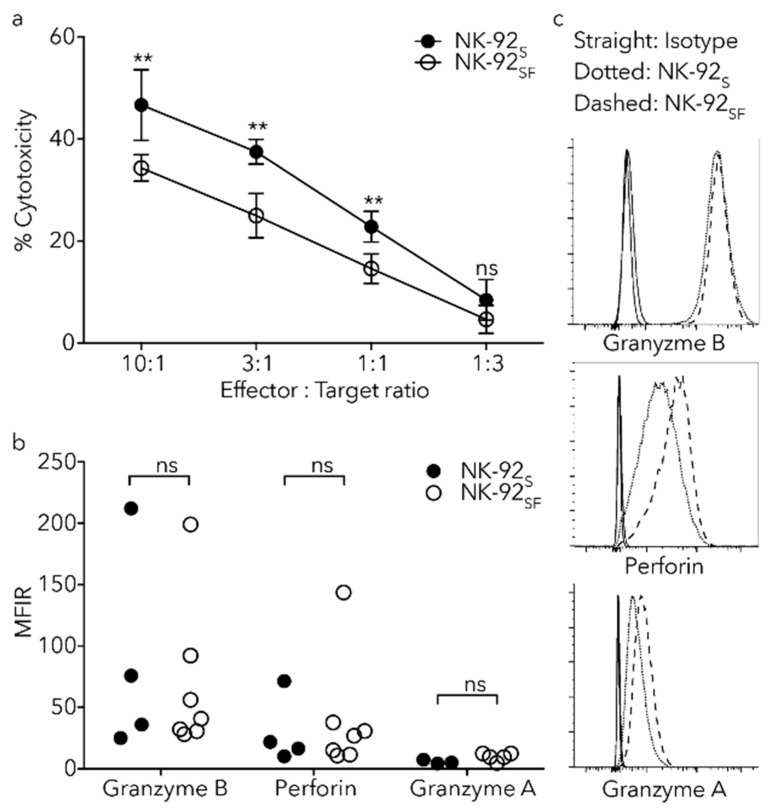
Reduced degranulation rate and cytotoxicity against K562 in NK-92_SF_ cells. (**a**) Chromium release assay, with K562 cells as target cells, show reduced cytotoxicity in NK-92_SF_ cells at designated effector:target ratios. Each data point represents the mean (+SD) of two independent assays performed in triplicates. (**b**) Load of Perforin, Granzyme A, and Granzyme B in NK-92_S_ and NK-92_SF_ cells as shown by gMFI ratio to isotype do not alter between culture conditions. Each bar represents the mean (+SD) of four independent assays performed in single or duplicate experiments. (**c**) Representative histograms showing indicated marker expression. Straight line shows isotype control, dotted line shows receptor expression on NK-92_S_ cells and dashed line shows receptor expression on NK-92_SF_ cells. Statistical significance (** *p* < 0.01). (**a**): 2way ANOVA and (**b**): Wilcoxon test was used.

As gradual serum reduction and long-term in vitro expansion of NK-92 cells did not significantly affect viability, cell proliferation or phenotype, we sought to assess their response against tumor cells. A standard ^51^Cr-release assay was performed by incubation of ^51^Cr labeled K562 cells together with NK-92 cells from different culture conditions for 4 h. We could identify that the capacity of cell-mediated lysis was reduced in the NK-92_SF_ cells (Figure 5a).

As short-term target-cell killing by NK cells is mainly due to the release of Granzyme B and Perforin [23], we examined their levels in NK-92_SF_ cells. Flow cytometry analysis showed no reduction of Granzyme A, Granzyme B, or Perforin in NK-92_SF_ cells (Figure 5b). Altogether, these findings indicate that serum reduction of NK-92 cells reduces cytotoxic response, while Perforin and Granzyme levels are not altered.

### 2.7. Killing Capacity of NK-92_SF_ Cells Is Recovered after Serum Addback

**Figure 6:**
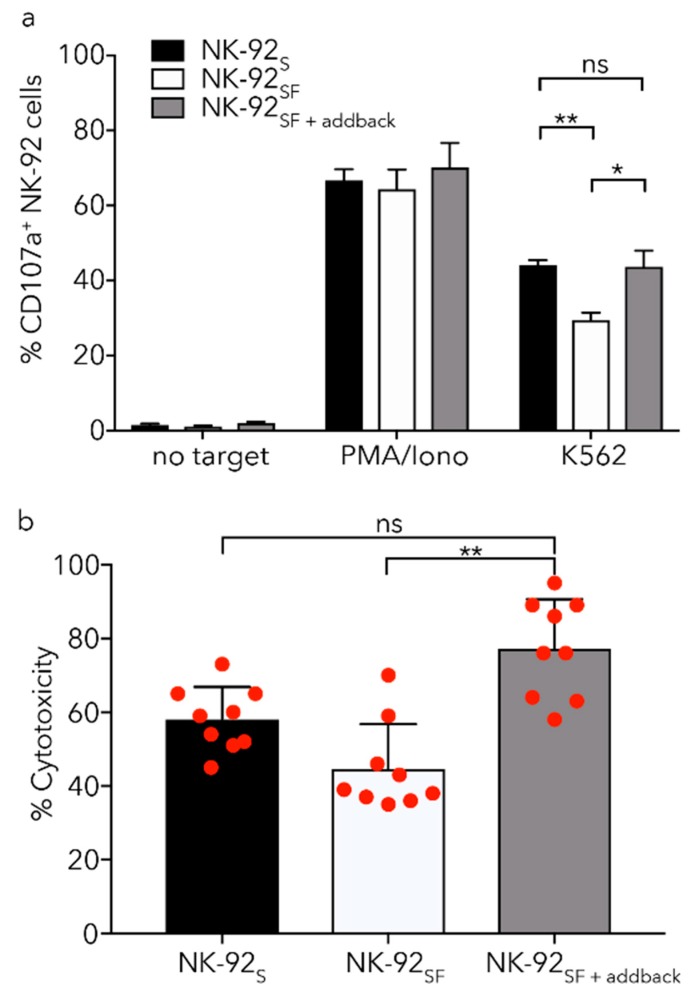

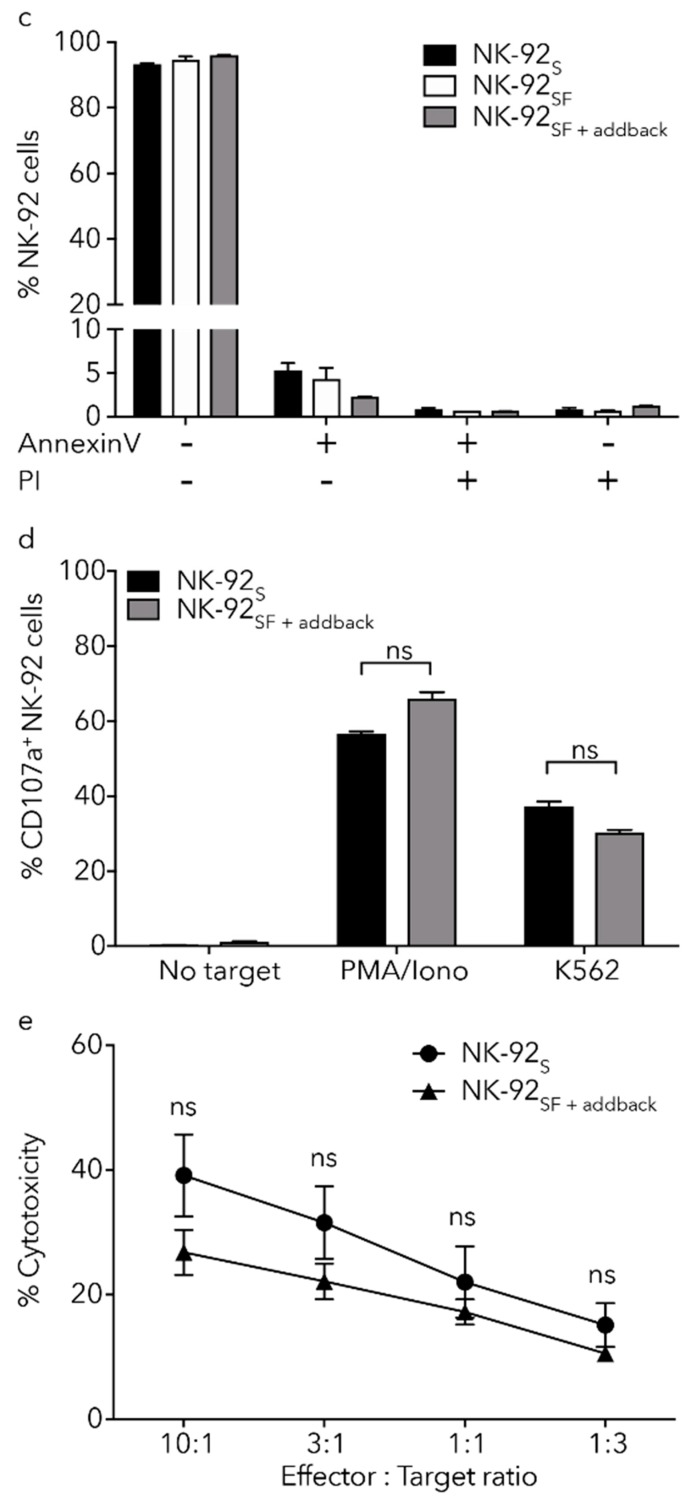
Addback of serum to NK-92_SF_ cells recovers killing capacity. (**a**) NK-92_SF_ show lower degranulation capacity against K562 target cells during a 5 h co-culture compared to NK-92_S_. NK-92_SF_ cells were cultured in 20% serum-containing media for 16 h (NK-92_SF+addback_) and degranulation was tested against K562 target cell line. (**b**) Re-introduction of 20% serum for 16 h (NK-92_SF+addback_) increases killing capacity in a 4 h chromium release assay significantly. (**c**) NK-92_SF_ cells are able to recover from freezing and thawing and show no altered viability 72 h post-thaw regardless of serum concentrations. Staining of CD56+ NK-92 cells with Annexin V and propidium iodide identified a proportion of live cells (Annexin V^−^/PI^−^), pre- (Annexin V^+^/PI^−^) and pro-apoptotic (Annexin V^+^/PI^+^) cells, as well as dead cells (Annexin V^−^/PI^+^). (**d**,**e**) NK-92 cells previously cultured with or without serum were kept in liquid nitrogen for long-term storage and then thawed and cultured for 16 h in complete medium (SCGM + 20% FBS). No significant difference in degranulation or cytotoxic capacity of NK-92_SF_ compared to NK-92S cells could be observed. For (**a**,**b**) and (**e**) each graph represents the mean (+SEM) of three independent assays performed in triplicates. (**c**,**d**) each graph represents the mean (+SEM) of two independent assays performed in duplicates. Statistical significance (* *p* < 0.05; ** *p* < 0.01). (**a**,**d**): Welch’s *t*-test, (**b**): Kruskal–Wallis test, (**e**): Two-way ANOVA were used.

In order to resemble the in vivo conditions with high serum concentration, long-term NK-92_SF_ cells were reintroduced to complete medium. Similar to the data from the cytotoxicity data we found that upon exposure to K562 cells, NK-92_SF_ cells had reduced CD107a expression compared to NK-92_S_ cells (Figure 6a). Interestingly, overnight incubation of NK-92_SF_ cells with complete medium led to an increased degranulation against K562 cells; comparable to that of NK-92_S_ cells (Figure 6a). Serum re-introduction also significantly increased the cytotoxic ability of NK-92_SF_ cells against K562 cells (Figure 6b).

### 2.8. Cryoprotection Has Similar Effects on NK-92_SF_ and NK-92_S_ Cells

For prospective use of these cells in adoptive immunotherapy, it was essential to investigate the viability and functional recovery of NK-92_SF_ cells that are thawed after long-term storage in liquid nitrogen. We could not detect any difference in viable and dead cell percentage of CD56^+^ NK-92_SF_ cells or NK-92_S_ cells when assessed three days after thawing (Figure 6c). As NK-92_SF_ cells had similar viability to NK-92_S_ cells, we next analyzed their functional recovery after freezing and thawing. NK-92_SF_ cells thawed and cultured in serum-containing medium overnight had similar degranulation (Figure 6d) and cytotoxic ability (Figure 6e) when compared to NK-92_S_ cells cultured with serum both before and after one freeze–thaw cycle. These data demonstrate that NK-92_SF_ cells have similar survival after freezing and thawing as NK-92_S_ cells, and that the degranulation and cytotoxic capacity are restored after overnight reintroduction of serum in the cell culture medium.

### 2.9. Lentiviral Transduction of NK-92 Cells Is Equally Efficient after Serum Reduction

**Figure 7:**
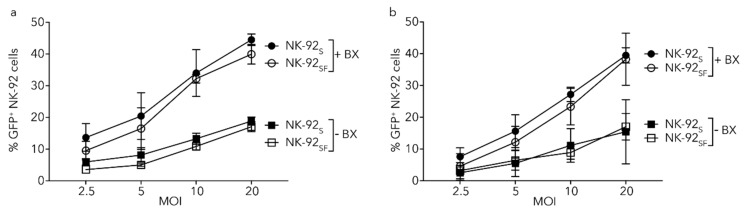
Serum-starvation of NK-92 cells does not change transduction efficiency with lentiviral vectors. CD56^+^GFP^+^ NK-92 cells three days after transduction with non-purified (**a**) or purified (**b**) LeGO-G2 virus in the absence or presence of BX-795. The graph represents the mean (+SEM) of two independent assays performed in duplicates. The LeGO-G2 virus is only introduced to express GFP, and the result was analyzed as GFP^+^ percentage three days post-transduction.

In order to see the effect of serum reduction on transduction efficiency, we tried transduction of NK-92_S_ and NK-92_SF_ at various virus multiplicity of infection (MOI), in the absence or presence of BX795, with non-purified (contains residual serum and factors secreted by HEK293FT cells) (Figure 7a) or purified (column-purified, serum-free) (Figure 7b) LeGO-G2 virus.

We observed that lentiviral gene delivery efficiency is comparable in between NK-92 cells grown with or without serum, showing expected gradual increase with increasing MOI both in the absence and the presence of BX795 (Figure 7a,b). Moreover, the enhancing effect of BX795 on lentiviral gene delivery showed accentuated results with increasing MOI for both conditions. Taken together, these results indicate that lentiviral gene delivery to NK-92_SF_ cells can be carried out as efficiently as cells grown with serum and this protocol can be adapted to future gene therapy approaches.

## 3. Discussion

In this study, we assessed the role of serum reduction on an NK cell line in order to understand the minimal serum requirements as well as the transcriptomic alterations that may compensate for such changes in cultivation. We observed that long-term cultivation of NK-92 cells was possible in a serum-free setting with no detected significant alteration in viability or proliferation.

Primary NK cells can regain their function against autologous tumor cells after ex vivo expansion and activation, but it is a major problem to achieve clinically feasible numbers of NK cells from healthy donors or patients [1]. NK cell expansion conditions are costly and not all expansions homogenously yield an appropriate amount of cells necessary for successful therapy. Serum composition and the factors that are important for efficient NK cell proliferation are convoluted with inter-individual variations, processing differences between different serum providers and batch to batch variations within each provider [24]. Even though the sera of various donors are generally pooled into batches in order to minimize and compensate for these variations, the concentration of growth factors and other serum components vary from batch to batch, leading to a variations in expansion efficiency [25].

In comparison to many classical media, such as DMEM and RPMI 1640, that are protein-free, the GMP-grade media used in this paper contained albumin (human plasma-derived), and insulin (human recombinant, yeast-derived). Albumin is the major protein in serum. The main functions of albumin in cell culture are antioxidant functions, maintaining pH, and binding and transport of important ligands such as lipids and amino [26]. Insulin helps cells utilize glucose and amino acids in the media. We have in this paper shown that these two proteins are sufficient for NK-92 cell culture in absence of serum.

The advantage of using a cell line for therapy is the predictable numbers of highly cytotoxic NK cells, which makes them suitable as an off-the-shelf cell therapy product for clinical use. With the method described in this paper, it is now possible to grow large numbers of NK-92_SF_ cells without losing their reactivity against target cells, especially when serum is added back into the culture. Based on the fold expansion rate (100-fold in two weeks) shown in this paper it would be possible to reach ideal numbers of cells to treat a 100 kg patient within a four week time period starting from 1 × 10^6^ cells.

Here, we show that NK-92_SF_ cells had a reduced cytotoxic response to K562 cells compared to NK-92_S_ cells. In an effort to mimic infusion of NK-92_SF_ cells, and, thus, exposure to serum in the patient, we have designed an in vitro addback experiment with overnight serum reintroduction. The addback experiment resulted in a restored cytotoxic response against standard NK cell targets. We could not detect any significant differences in the expression of NK cell markers or adhesion receptors on NK-92 cells between the culture methods. This implies that the differences in outer appearance and functionality must rely on other mechanisms.

One of the major mechanisms of NK mediated tumor cell killing is degranulation, i.e., the release of Granzyme B and Perforin by the effector cell [23]. Our results showed no differences in cytolytic protein content nor activating receptor expression between NK-92_SF_ and NK-92_S_ cells. Furthermore, NK-92_SF_ cells proliferated at a similar rate when compared to NK-92_S_ cells.

Interestingly, comparative gene expression profiling of serum reduction indicated that a gradual decrease of serum may lead to MYC-driven retention of proliferation and a significant activation of immune response pathways. This may suggest that intermittent serum reduction might be a feasible strategy for transiently skewing gene expression in order to achieve a more proliferative and activated product in a homogenous manner. We could show that the interferon signaling pathway, as well as the antigen presentation pathway, was highly upregulated in NK-92_SF_ cells. Upregulation of those pathways is likely an indirect sign of potentially increased activation status due to the lack of serum and resulting stress [27]. Activated NK cells are able to effectively stimulate T cells and can have a role similar to antigen-presenting cells [28]. This effect will be triggered by upregulation of MHC class II molecules together with multiple ligands to TCR costimulatory molecules [28]. Additional studies, using primary NK cells as well as other NK cell lines, are warranted to prospectively elucidate the role of individual pathways in order to optimize a potential NK cell therapy product. Since a 3-week serum reduction protocol is not directly adaptable to primary NK cell expansion protocols due to the time required, a detailed understanding of serum factors that are critical for NK cell homeostasis and proliferation need to be assessed in future studies.

There are currently multiple ongoing clinical trials with the NK-92 cell line in China, Germany, and in the USA (clinicaltrials.gov, NCT03387085, 03387111, 03586869) [18,29,30] and its role for preclinical studies is becoming clearer [31]. We believe that our observations reported here can provide some helpful insights and decreased manufacturing costs for future studies.

In summary, we have demonstrated that the gradual reduction of serum leads to stable growth of NK-92 cells. The ability to restore the cytotoxic capacity of NK-92_SF_ cells subsequent to serum addback and no significant alterations in degranulation after freeze–thaw cycle suggests that a robust and affordable expansion procedure is possible and potentially as feasible as current standard clinical manufacturing protocols.

## 4. Materials and Methods

### 4.1. Cell Lines and Cell Culture

The human NK cell line NK-92 was purchased from American Type Culture Collection (ATCC, Manassas, VA, USA, catalog number: CRL-2407^™^). Cells were initially thawed in a stem cell growth medium (CellGro; CellGenix, Freiburg, Germany) with 20% heat-inactivated FBS (Gibco, Life Technologies, Carlsbad, CA, USA) and 1000 U/mL of Proleukin (Novartis, Basel, Switzerland) or IL-2 (Miltenyi, Bergisch Gladbach, Germany) when available. To evaluate NK cell activity, the human erythroblast cell line from a chronic myelogenous leukemia patient K562 (LGC Promochem/ATCC, Manassas, VA, USA) was used as a target in degranulation assays and ^51^Cr-release assay. K562 cells were cultured with RPMI, GlutaMAX 1640 (Gibco) supplemented with 10% FBS. Cells were incubated at 37 °C and 5% CO_2_ with a humidity of 95% and cell numbers were determined every second day by Trypan Blue staining. NK-92 cells cultured in standard conditions were kept in between 0.3–1.0 × 10^6^ cells/mL density, while NK-92_SF_ cells were cultured at a concentration of 0.5–1.5 × 10^6^ cells/mL and supplemented with 1000 U/mL IL-2 5 days per week in order to obtain optimal cell proliferation. All cell culture has been conducted in a BSL2 environment under strict antibiotic-free conditions. HEK293FT cell line (Thermo Fisher Scientific, Waltham, MA, USA) is an altered version of adherent human embryonic kidney cells, expressing SV40 large T antigen that provides high titer lentivirus production due to high expression of viral RNA. Both virus production and titration experiments were optimized with this cell line. HEK293FT cells were cultured in Dulbecco’s Modified Eagle Medium (DMEM, Gibco) Glutamax with 10% FBS (Gibco), 1 mM l-glutamine (Sigma-Aldrich, St. Louis, MO, USA), 1 mM sodium pyruvate solution (Sigma-Aldrich), and 0.1 mM MEM non-essential amino acids (Sigma-Aldrich).

### 4.2. Serum Reduction

In order to adapt NK-92 cells to serum-free culture conditions, the serum concentration was successively reduced in culture. Culture medium was completely changed and fresh medium with a new serum concentration was added to NK-92 cells weekly by decreasing the serum concentration by half (20%, 10%, 5%, and 0% FBS). Cells cultured with 0% FBS were kept at a density of 0.5–1.0 × 10^6^ cells/mL.

### 4.3. Flow Cytometry

NK cell phenotype was analyzed with fluorochrome-conjugated antibodies against CD2 (RPA-2.10), CD11a (HI111), CD11b (ICRF44), CD27 (M-T271), CD44 (G44-26), CD57 (NK-1), CD62L (DREG-56), CD107a (H4A3), DNAM1 (DX11), KI-67 (B56), NKp44 (p44-8.1) NKp46 (9E2), NKp30 (p30-15), NKG2D (1D11), Siglec 9 (E10-286) (BD Biosciences, Franklin Lakes, NJ, USA); NKG2C (134591) (R&D Systems, Abingdon, UK); CD2 (TS1/8), CD56 (HCD56), NKp80 (5d12), CD160 (BY55), CD161 (HP-3G10), CD319 (CRACC) (162.1), and CD352 (NTBA) (NT-7) (Biolegend, San Diego, CA, USA); CD244 (2B4) (C1.7) (Beckman Coulter, Brea, CA, USA); NKG2A (REA110), Siglec 7 (REA214) (Miltenyi) and handled according to the manufacturers’ protocol. For intracellular staining of Granzyme A (CB9) (Biolegend), Granzyme B (GB11), and Perforin (dG9) (BD Biosciences), cells were washed with PBS followed by fixation and permeabilization with Cytofix/Cytoperm (BD Biosciences) and incubated for 20 min. Following fixation and permeabilization, cells were washed and stained in Permwash according to manufacturer’s protocol. All cells were acquired by a BD LSR Fortessa flow cytometer (BD Biosciences) and analyzed using FlowJo software (FlowJo LLC, Ashland, OR, USA).

### 4.4. RNA Preparation and Sequencing Data Analysis

Cells were frozen in RLT-buffer and ß-Mercaptoethanol (Qiagen, Venlo, The Netherlands) and RNA was extracted according to Illumina (San Diego, CA, USA) instructions for the TruSeq RNA Library Prep Kit. All samples were processed and sequenced on a HiSeq 2500 (Illumina, San Diego, CA, USA). Each sample received between 23 and 27 million reads. The quality of the reads was high and Phred scores were significantly greater than 30 (99.9% base calling accuracy).

After quality control, alignment was performed using the STAR aligner and transcript abundance by HTSeq. Raw transcript counts were normalized with EdgeR (Bioconductor), using the method where normalization factors are calculated to scale the raw library sizes, using the method ‘TMM’ (Trimmed Mean of M-values). A negative binomial generalized log-linear model (GLM) is fitted to the read counts for each gene or transcript and a likelihood ratio test is conducted for one or more coefficients in the linear model. The differential gene expression tests were implemented in EdgeR and fold change, *p*-values, and FDRs (false discovery rates) were reported. Comparisons of gene expression profiles were subsequently generated and uploaded into Ingenuity Pathway Analysis (IPA).

### 4.5. Apoptosis, Cell Cycle Analysis, and Proliferation

For identification of pre- and pro-apoptotic and necrotic cells in culture the NK-92 cells were stained with Annexin V and propidium iodide according to manufactures protocol (FITC Annexin V Apoptosis Detection Kit I, BD Biosciences). Frozen cells were thawed and analyzed immediately. For proliferation analysis, the cells were frozen in 70% ethanol for at least 2 h and then stained with KI-67. CFSE was used for investigating proliferation over a 4 day period. NK-92 cells were labeled in 2 μM CFSE (BD Biosciences) for 8 min in the dark at room temperature and then cultured as described above for 4 days. CFSE intensity was measured with a LSR Fortessa flow cytometer and analyzed using FlowJo.

### 4.6. Karyotyping

Demecolchicine (Sigma-Aldrich), at a final concentration of 0.005 μg/mL, was added to the cell cultures overnight to arrest the cells in a mitotic state. After detaching the cells they were treated with a hypotonic, 0.56%, potassium chloride solution for ten minutes, centrifuged and resuspended in a fixation solution, acetic acid:methanol, 1:3. After another round of fixation cell suspension was spread out on wet, cold slides. The slides were flame dried to improve the spreading of the chromosomes. Conventional Q-banding technique was used according to the International System for Human Cytogenetic Nomenclature, ISCN 2009, and cytogenetic analysis was performed with a Cytovision Image analysis system (Applied Imaging, Sunderland, UK). 20 metaphases were analyzed from each culture. The cells of the second production of NK-92 serum-starved cells were analyzed as described above. 23 metaphases were analyzed of each culture.

### 4.7. Degranulation and Cytotoxicity Assay

To analyze the NK-92 cells ability to degranulate against the K562 cell line, cells were cultured at a density of a 1 × 10^6^ cells/mL in a 96 well plate and incubated alone or with K562 at a ratio of 1:1 in 200 μL or with phorbol 12-myristate 13-acetate (PMA) and ionomycin (0.5 μg/mL, Sigma-Aldrich), together with CD107a antibody for 4–6 h. After 1 h of incubation, GolgiStop (BD Biosciences) was added to cultures to inhibit protein transportation. Subsequently, cells were stained for CD56 for 30 min at 4 °C. Degranulation was analyzed with a LSR Fortessa flow cytometer. Additionally, NK-cell cytotoxicity was measured in a ^51^Cr-release assay against K562 cells [32]. Briefly, K562 cells were labeled with 100 uCi ^51^Cr (PerkinElmer, Waltham, MA, USA) for 1 h at 37 °C, NK cells were mixed with the labeled K562 cells at different effector:target ratios and incubated for 4 h. For Figure 5a and Figure 6e, supernatant (70 μL) was transferred into 4 mL sample tubes and counted using a 2470 WIZARD2 automatic gamma counter (PerkinElmer). For the ^51^Cr-release assay in Figure 6b, 25 μL of the supernatant was transferred to LumaPlate-96 and consequently analyzed with a MicroBeta2 (PerkinElmer).

### 4.8. Lentivirus Production

The protocol for the production of lentiviral vectors was adapted from Sutlu et. al. 2012 [33]. In short, HEK293FT cells were seeded out 8–10 h prior to transfection with a 3rd generation lentiviral plasmid mix using a Calcium Phosphate precipitation kit (Sigma-Aldrich). Lentiviral particles were harvested 24 and 48 h after a medium change and were filtered through a 0.45 μm filter and stored at −80 °C without repeated freeze–thaw cycles. Virus titer was obtained by transducing HEK293FT cells with a serial dilution of viral supernatant and measuring transgene expression by flow cytometry 3 days post-transduction. Serum-free virus batches were obtained by purifying 40 mL viral supernatant using Vivapure LentiSELECT 40 kit (Sartorius, Göttingen, Germany) according to the manufacturer’s protocol.

### 4.9. Lentiviral Transduction

Lentiviral transduction of NK-92 cells was prepared with 2.5 × 10^5^ cells per well in 24-well tissue culture plates, at specified multiplicity of infection (MOI) in the presence or absence of inhibitor BX795 (Invivogen, Toulouse, France) (3 to 6 μM final concentration was used), supplied with 1000 U/mL IL-2 and 8 μg/mL Protamine Sulfate (Sigma-Aldrich) for 6 h. After culturing cells in virus-containing media for the specified time, cells were centrifuged for 5 min at 300× *g*. Virus-containing supernatant was completely removed and cells were cultured in their regular growth media for 72 h before green fluorescent protein (GFP) expression was checked using flow cytometry.

### 4.10. Statistical Analysis

For the preparation of graphs and statistical analysis, Graph-Pad Prism (GraphPad Software Inc., La Jolla, CA, USA) was used.

## 5. Conclusions

NK-92 cells are able to grow under serum-free conditions, leading to a robust and affordable expansion procedure for potential clinical manufacturing.
